# Estimation of the average molecular weight of microbial polyesters from FTIR spectra using artificial intelligence

**DOI:** 10.1007/s44211-025-00780-2

**Published:** 2025-05-08

**Authors:** Peter Polyak, Paweł Chaber, Marta Musioł, Grażyna Adamus, Marek Kowalczuk, Judit E. Puskas, Miroslawa El Fray

**Affiliations:** 1 Department of Food, Agricultural and Biological Engineering, College of Food, Agricultural, and Environmental Sciences, The Ohio State University, 1680 Madison Avenue, Wooster, 44691 USA; 2 Centre of Polymer and Carbon Materials, Polish Academy of Sciences, 34, M. Curie-Skłodowska St, 41-819 Zabrze, Poland; 3 Department of Polymer and Biomaterials Science, West Pomeranian University of Technology in Szczecin, al. Piastow 45, 70-311 Szczecin, Poland; 4 Centre of Advanced Materials and Manufacturing Process Engineering, West Pomeranian University of Technology, Szczecin, al. Piastow 45, 70-311 Szczecin, Poland

**Keywords:** IR spectroscopy, Microbial polyester, PHB, ANN, Machine learning

## Abstract

In this paper, we present a method for calculating the average molecular weight of microbial polyesters using Fourier transform infrared spectroscopy (FTIR) data as input. FTIR spectra provide the necessary quantitative information, as the impact of chain ends on the spectra is influenced by the average molecular weight of the polymer. Since FTIR data can be collected rapidly and is available in abundance, it serves as an ideal input for machine learning algorithms, such as artificial neural networks. The robustness and reliability of the model are improved by designing the neural network to use absorbance ratios instead of absolute absorbances as input. We also propose a new feature selection method that facilitates the identification of absorbance ratio regions best suited to serve as input for the neural network. Our approach ensures that variations in sample preparation do not compromise the accuracy of the model. The proposed computational method is demonstrated using a microbial polyester [poly(3-hydroxybutyrate), PHB], which is a biopolymer natively synthesized by multiple bacterial strains. Although the computational method has been tested with PHB, the underlying concept can be extended to other polymers. To facilitate broader application, a step-by-step guide for developing similar models is also provided.

**Graphical abstract:**

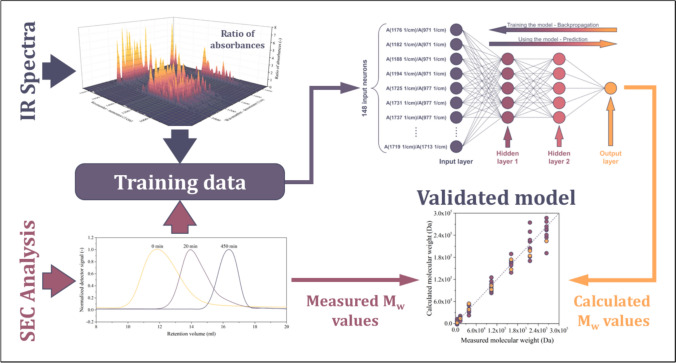

**Supplementary Information:**

The online version contains supplementary material available at 10.1007/s44211-025-00780-2.

## Introduction

Sustainability and the transition towards a circular economy are key priorities in contemporary polymer research. Among biopolymers, microbial polyesters have emerged as promising candidates due to their renewable production and recyclability. While the goal is always the circularity of material usage, the optimal recycling method depends on the type of the plastic waste. The recycling of some polymers, such as polyolefins, is more energy-efficient if only mechanical recycling takes place. In other cases, chemical recycling that involves complete depolymerization is more beneficial [1]. Microbial polyesters can be recycled both mechanically and chemically, and offer a fully sustainable alternative to fossil resource-based polyolefins [2, 3]. These biopolymers are produced by bacterial strains that utilize them as intracellular material and energy storage [4, 5]. Microbial polyesters can be biosynthesized using many types of renewable material sources as nutrients for the bacterial culture. The most important ones are carbohydrates [6–8] and lipids [9, 10] produced by plants. To improve production efficiency and to reduce associated costs, several research projects were carried out in the past few decades that targeted the fermentation of microbial polyesters using waste materials and byproducts. These sources include agricultural waste [11], waste from the food industry [12], and byproducts of the paper industry, such as lignin [13]. Recent studies have shown that glycerol can also be used as a substrate for polymer-producing bacteria [14–16]. Since glycerol is abundantly available as a side product of biofuel synthesis, microbial polyester production would perfectly complement the expanding biofuel industry [14–16].

Despite all these advantages and the potential of microbial polyesters to substitute their fossil resource-based counterparts (most importantly, polyolefins, such as polyethylene and polypropylene), the market share of these biopolymers is still comparatively small. The main reasons that impede the success of microbial polyesters include the multidisciplinary knowledge required for their cost-efficient production (genetic engineering [17], biotechnology [18], biorefinery [19], and polymer processing [20–23]). It is also important to find conditions yielding high molecular weights to have good mechanical performance [24–28]; therefore, gaining accurate information on this parameter is of utmost importance.

Although multi-detector size exclusion chromatography (SEC) equipped with multi-angle laser light (MALS) detector is the best method to measure absolute molecular weights, it requires expensive instrumentation. Furthermore, SEC with only relative methods [polystyrene or poly(methyl-methacrylate) standards and Mark-Houwink-Sakurada MHS shifts] have been reported for microbial polyesters [29–32]. In order to overcome these limitations and to facilitate the rapid and cost-efficient measurement of the average molecular weight of microbial polyesters, we would like to propose an entirely different approach. Our concept is based on infrared spectroscopy (IR), as IR data can be collected routinely, without requiring high-end instrumentation (such as SEC systems) or involving labor-intensive sample preparation. The computational procedure relies on the correlation between the IR data and the average molecular weight. Information about the length of the macromolecular chains can be obtained by assessing the characteristics of the peaks associated with resonances of groups located in the middle of the chains [33]. The shorter the macromolecular chains, the larger the relative amount of end groups. Therefore, the average molecular weight influences the ratios of IR absorbances. In conclusion, IR data carries the targeted quantitative information and can be used as input for predictive mathematical models that are capable of handling vast amounts of input data, such as machine learning algorithms.

Even if the input dataset carries the targeted quantitative information, using the entire dataset to build machine learning models often results in poor performance. A prerequisite of building reliable and accurate models is the selection of input variables that correlate with the predicted variable, *i.e.*, the output of the model. This step is generally referred to as ‘feature selection’ or ‘variable selection’ and is intensively studied due to its importance. Several feature selection methods have already been proposed in the literature. The most relevant ones are filter methods (*e.g.*, interval partial least squares, iPLS [34]), wrapper methods (*e.g.*, recursive feature elimination, RFE [35]), embedded methods (e.g., least absolute shrinkage and selection operator, LASSO [36]), and evolutionary and metaheuristic methods (*e.g.*, genetic algorithms [37, 38]).

Notable examples of machine learning models that could rely on IR data and the feature selection methods cited in the previous paragraph are artificial neural networks (ANN) [39–41]. In our concept, the IR spectra of microbial polyesters are used as an independent variable of the ANN model, whereas its dependent variable is the estimated average molecular weight. Like in the case of all machine learning models, the construction of a reliable, ANN-based model requires large volumes of accurate training data [42, 43]. IR spectroscopy is an ideal source of training data in this case, as it can be collected rapidly and routinely. While previous models relied on conventional methods, such as Partial Least Squares (PLS) regression [44–46], we propose the implementation of the concept of neural networks. Although ANNs have already been used in multiple engineering fields [47–50], we are the first to report their application for IR data-based computation of average molecular weights.

## Materials and methods

### Materials

Poly(3-hydroxybutyrate) (PHB) was kindly provided by Biomer (Krailing, Germany). Before it was used as a starting material for the alcoholysis, PHB was purified by precipitation from chloroform in n-hexane. The precipitated solid was filtered off, vacuum-dried, and analyzed by size exclusion chromatography (SEC, see “SEC analysis”.). Chloroform, n-hexane, 1,2-dichloroethane, p-toluene sulfonic acid, n-butanol, and ethanol were purchased from VWR (Part of Avantor, Gliwice, Poland). All reagents were used as received.

### Preparation of samples with different average molecular weights

A series of samples were obtained by acid-catalyzed alcoholysis of the Biomer PHB. 4 g of PHB was dissolved in 50 cm^3^ of boiling 1,2-dichloroethane; then, p-toluene sulfonic acid (0.2 g) and n-butanol (5.2 cm^3^) were added to the reaction flask. The reaction was stirred at 80 °C for 7.5 h. The first aliquot of the reaction mixture (3.5 cm^3^) was taken 1 min and 15 s after adding n-butanol. This aliquot was then poured into 35 cm^3^ of cold ethanol. The precipitated white solid was filtered, washed thoroughly with water, and dried in a vacuum oven for one day. In this way, a sample marked as PHB1 was obtained. The samples labeled as PHB2, PHB5, PHB10, PHB20, PHB40, and PHB450 were obtained by taking aliquots of the reaction mixture at 2.5, 5, 10, 20, 40, and 450 min, respectively, after starting the alcoholysis. These aliquots were purified as described in the case of the PHB1 sample.

### NMR analysis

^1^H NMR spectra were recorded using a Bruker-Avance II 600 MHz with Ultrashield Plus Magnets. The ^1^H spectra were run with CDCl_3_ as the solvent and using tetramethyl silane as an internal standard. NMR spectra were obtained with 64 scans and 2.65 s acquisition time. The time domain size was 65,536 points, and four dummy scans were performed before collecting the NMR data. The results were processed and evaluated using SpinWorks 4.2.9 and Origin 9.2.257.

### SEC analysis

The samples for SEC analysis were prepared by dissolving the polymer in chloroform at a concentration of 0.3% w/v and passed through the solvent delivery system Nexera HPLC/UHPLC Pump—LC-40D XR (Shimadzu, Kyoto, Japan), at 35 °C with a flow rate of 1 mL/min. This process involved using two Mixed C Styragel (Agilent Technologies, Inc., Santa Clara, CA, United States) columns with a mixed bed (M_w_ = 200–2,000,000) for analysis and a Shodex RI-101 refractive index detector (SHOKO Scientific Co. Japan). Narrow molar-mass dispersity polystyrene (PS) standards from EasiCal^®^ Pre-prepared Calibration Kits provided by Agilent Technologies, Inc. were used to create a calibration curve. PS equivalent M_w_ data was used to create the predictive model for the convenience of future use since MHS shifts do not result in significantly different M_w_ values [51].

### IR analysis

13.44 mg of polymer samples were added to 3 cm^3^ chloroform. The mixtures were shaken until the samples completely dissolved, forming a homogeneous solution with a concentration of 0.3 m/m%. 50 μL of the solutions were dropped on the surface of an IR-transparent carrier layer (KBr pellet) and dried. The resulting polymer films had a thickness of 1.407 μm, which was calculated from the area of the KBr pellet and the mass of the polymer. The KBr pellets, now carrying the polymer film, were placed in the metal frame of the IR spectrophotometer and analyzed in transmission mode. Spectra were collected using a Jasco FT-IR-6700 instrument that was configured to measure 4000–500 1/cm range with 1 1/cm resolution. In the case of each sample, 64 scans were accumulated, and each sample was measured ten times.

### Computational method

Modeling was based on ANNs using fully connected neural networks with biases. The concept of fully connected ANNs was implemented using software developed by our research team. All code was written in MATLAB integrated development environment. The version of MATLAB was R2024a Update 6 (24.1.0.2697110); the Integrated Development Environment was running in the MathWorks cloud (*i.e.*, Online MATLAB was used). While writing the code, three toolboxes (Statistics and Machine Learning Toolbox, Curve Fitting Toolbox, and Deep Learning Toolbox) were used. The performance of the code was quantified by MATLAB’s built-in Performance Timing Functions.

## Results and discussion

### NMR measurements

**Fig. 1:**
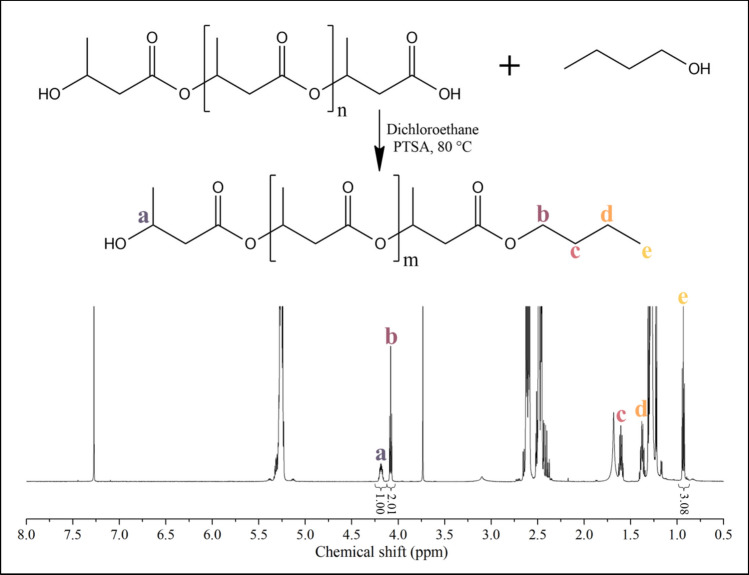
Scheme of the alcoholysis and the NMR spectrum of the ‘PHB450’ sample

Figure 1 shows the expected end group structure after the alcoholysis and a representative ^1^H NMR spectrum of the product. The integral ratios of proton ‘a’ to protons ‘b’ and ‘e’ are 1:2:3, demonstrating successful alcoholysis.

### SEC measurements

**Fig. 2:**
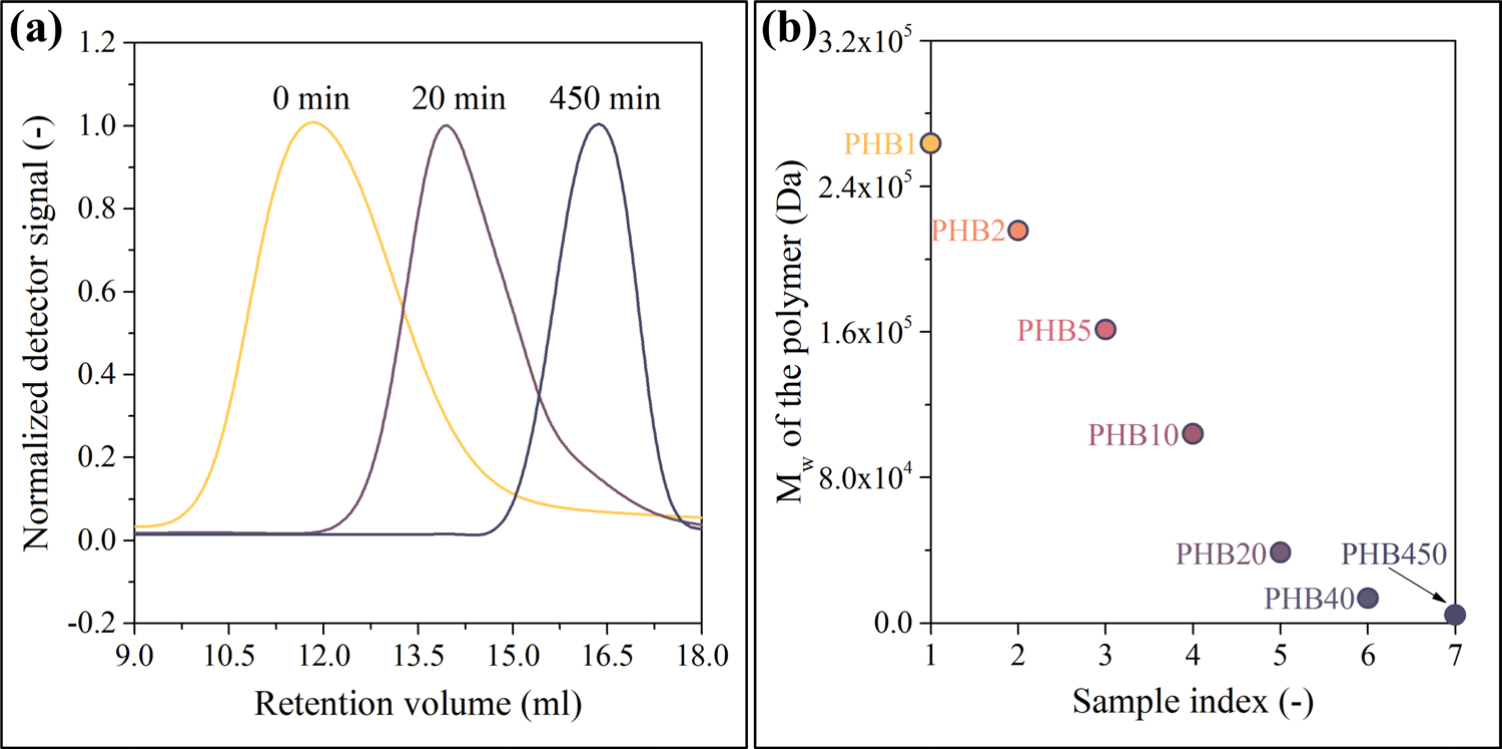
Chromatograms shifting to higher retention volumes (**a**). As presented in **b**, the M_w_ of the samples are distributed evenly and span a large M_w_ region

SEC analysis of the purified Biomer PHB yielded M_w_ = 774 kDa with Đ = 2.09. Figure 2a shows the SEC traces of the starting PHB, the 20-min, and the 450-min samples. The alcoholysis reduced the molecular weights while the polydispersity remained around Đ = 2. Figure 2b shows data for the whole series where the sample indices 1–7 stand for PHB1, PHB2, PHB5, PHB10, PHB20, PHB40, and PHB450. PHB450 had M_w_ = 4060 Da. From this value, M_n_ = 2030 Da can be calculated using Đ = 2.

Figure 2b reveals that our experimental method enables the creation of samples that are evenly distributed across the investigated molecular weight range and span the region having industrial potential. This is an especially important factor because the model will be able to provide accurate results only in the molecular weight range that is covered by the samples serving as a source of training data.

### IR measurements

**Fig. 3:**
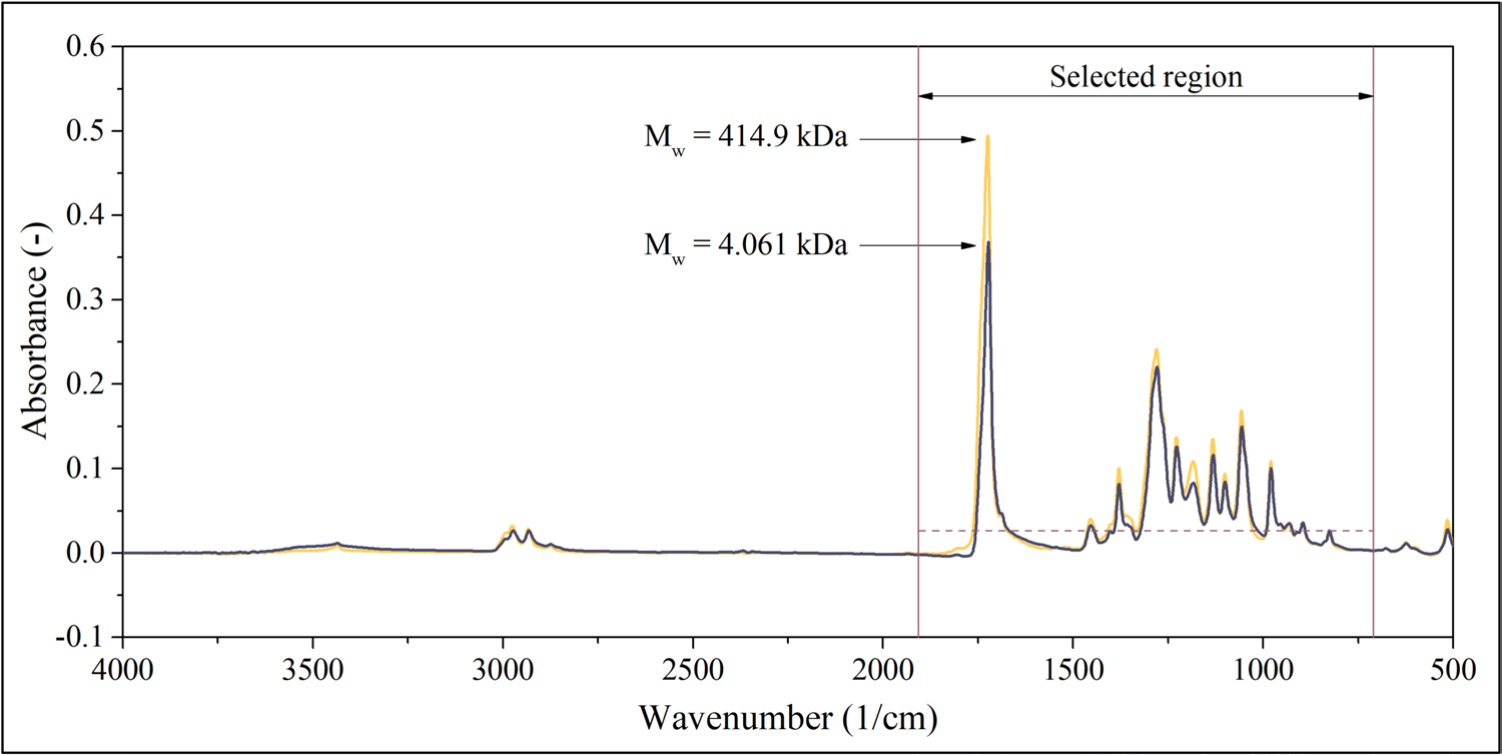
IR spectra of the first and last (PHB1 and PHB450) samples and the region selected as an input for the model. Absorbances will be used in further calculations if they exceed a threshold visualized as a dashed line

Figure 3 displays the IR absorbance values obtained by averaging 10 spectra of the first (PHB1) and last (PHB450) samples (as mentioned in the experimental section, each sample has been measured 10 times). The peak of the highest amplitude at 1724 1/cm can be attributed to the carbonyl groups, whereas the resonances of the C–O–O groups of the ester bonds appear in the 1000–1300 1/cm wavenumber range. Outside the 800–1800 1/cm region, only the 2800–3000 1/cm wavenumber range contains peaks that can be detected with a considerable signal-to-noise ratio. These resonances can be attributed to the stretching and bending of the C–H groups. As the signal-to-noise ratio of these peaks might not be sufficiently large, only the 800–1800 1/cm wavenumber range will be used as a source of input for the model.

### IR data processing

Figure 3 shows that the amplitudes of absorbance peaks seem to depend on the average molecular weights, but the differences are quite small. The weak correlation between the absolute absorbances and the average molecular weight suggests that conventional, normalization-based data processing methods (such as the calculation of Standard Normal Variate (SNV), presented in Supporting Information file SI1) might not be optimal in this case. Therefore, the mathematical model will not be based on the correlation between normalized absorbances and the average molecular weight. Rather, absorbance ratios will serve as input. The goal of using ratios instead of absolute absorbances is to address inconsistencies in sample preparation (creation of films with the same thickness on the surface of the carrier) and to foster reproducibility. This method can be considered a generalized implementation of the concept of internal standards. Instead of introducing one additional component of a known concentration (‘internal standard’) to the polymer sample and normalizing all amplitudes with the peak height of the internal standard, the ratios of all peak heights in all possible combinations are computed. Accordingly, the next step of processing the IR data is the calculation of all possible ratios of absorbances collected in the 800–1800 1/cm wavenumber range. The ratios are computed as presented in Eq. 1. Figure 3 shows that at some wavenumbers, the absorbances are either very close to or equal to zero. Therefore, the ratios are not calculated if the absorbances fall below a threshold. This threshold value (*A* = 0.03) is graphically represented by a dashed line in Fig. 3.

1 $$R({\nu }_{1},{\nu }_{2})=\frac{A({\nu }_{1})}{A({\nu }_{2})}$$

**Fig. 4:**
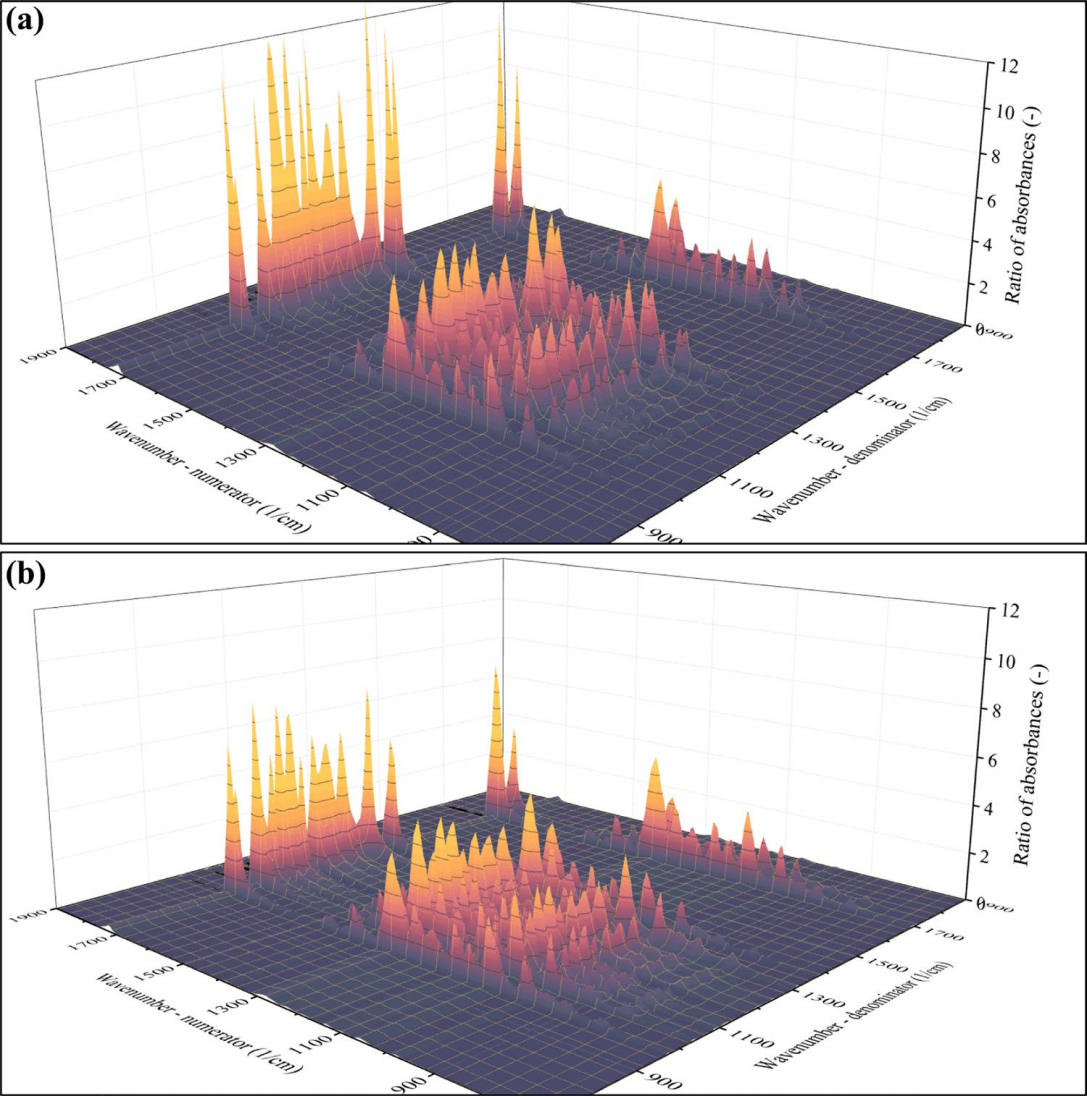
Appearance of the *R*(*ν*_1_, *ν*_2_) surface functions calculated as a ratio of absorbances. The spectrum that formed the basis of the calculation was that of the M_w_ = 263.7 kDa (t = 1 min, **a**) and M_w_ = 4060 Da (t = 450 min, **b**) sample

In Eq. 1, *R* is the ratio of absorbances, and *ν*_*1*_ (Greek nu) marks the wavenumber at which the absorbance in the numerator has been collected. Likewise, *ν*_*2*_ denotes the wavenumber corresponding to the absorbance in the denominator. Lastly, *A* marks the wavenumber-dependent absorbance. Since *R* has two independent variables (*ν*_*1*_ and *ν*_*2*_), it is a surface function that has been calculated in the case of each individual spectra. The appearance of the *R*(*ν*_1_, *ν*_2_) surface function computed using the spectra of the t = 1 min sample (M_w_ = 263.7 kDa) is shown in Fig. 4a. Even without any in-depth numeric analysis, the difference between the surface functions belonging to the M_w_ = 263.7 kDa (t = 1 min, Fig. 4a) and the M_w_ = 4060 Da (t = 450 min, Fig. 4b) sample is clearly noticeable.

**Fig. 5:**
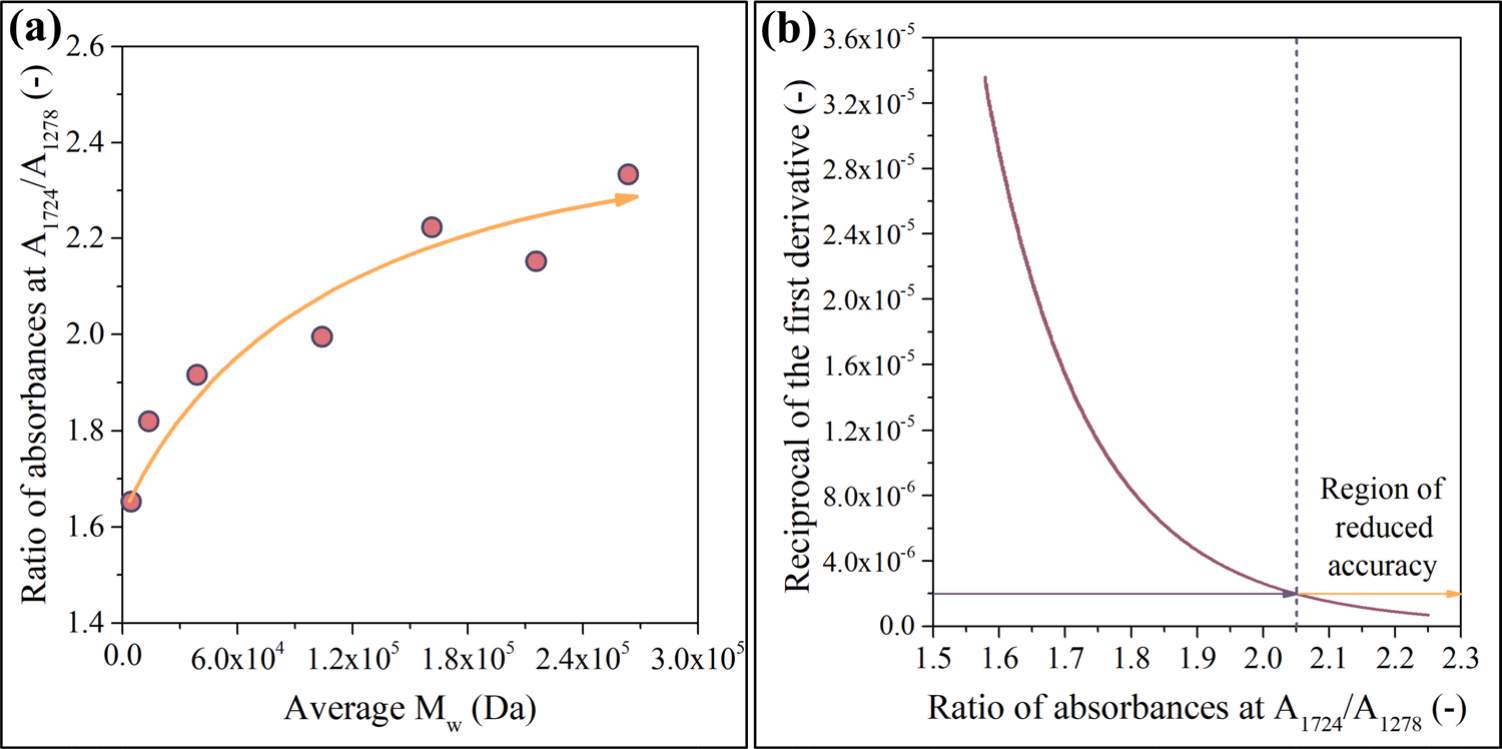
Ratio of absorbances (A_1724_/A_1278_) plotted against M_w_ (**a**). Due to the saturation-like characteristics shown in **a**, the model is expected to become less accurate at high M_w_ values (**b**)

The difference suggests that the ratios strongly depend on the average molecular weight of the samples. Note that the maximum of absorbance ratios plotted in Fig. 4a exceeds 12 (–) for the M_w_ = 263.7 kDa sample, whereas it does not reach 8 (–) in the case of the M_w_ = 4060 Da sample (Fig. 4b). This disparity highlights that the ratios of absorbances correlate with the average molecular weight of the sample. In the next step, this correlation will be quantitatively characterized as a function that describes the relationship between independent and dependent variables. In this context, the independent variables are available as a sequence of *R*(*ν*_1_, *ν*_2_) surface functions, whereas the dependent variable is one scalar (the M_w_ of the polymer). Supplementary video SI2 demonstrates the correlation between the above-mentioned sequence of *R*(*ν*_1_, *ν*_2_) surface functions and the average molecular weight. The graphical representation of this correlation can be simplified by selecting only one point on the *R*(*ν*_*1*_, *ν*_*2*_) surface. For example, the ratio of the two largest peaks in the spectrum (carbonyl vibration at 1724 1/cm and one of the peaks belonging to the C–O–O vibration at 1278 1/cm) is worth investigating separately; see Fig. 5a.

Figure 5a shows that the A(1724 1/cm)/A(1278 1/cm) ratio correlates positively and nonlinearly with M_w_. As the correlation asymptotically reaches a limit, the model is expected to become less accurate at high M_w_ values. The second important factor that must be paid attention to in Fig. 5a is the designation of the axes. The construction of the model is based on the creation of samples with various molecular weights and their analysis using IR. Therefore, plotting molecular weights on the horizontal axis and absorbance ratios on the vertical axis appears to be the most straightforward way of presenting the results. However, the neural network will take ratios of absorbances as independent variables and will output M_w_ values. Thus, modeling is based on the inverse of the correlation function shown in Fig. 5a**.** Due to the saturation-like characteristics presented in Fig. 5a, the inverse function (discussed in detail in Supporting Information file SI1) asymptotically converges to infinity. This convergence suggests that the model will lose accuracy in the region of high molecular weights (see Fig. 5b).

**Fig. 6:**
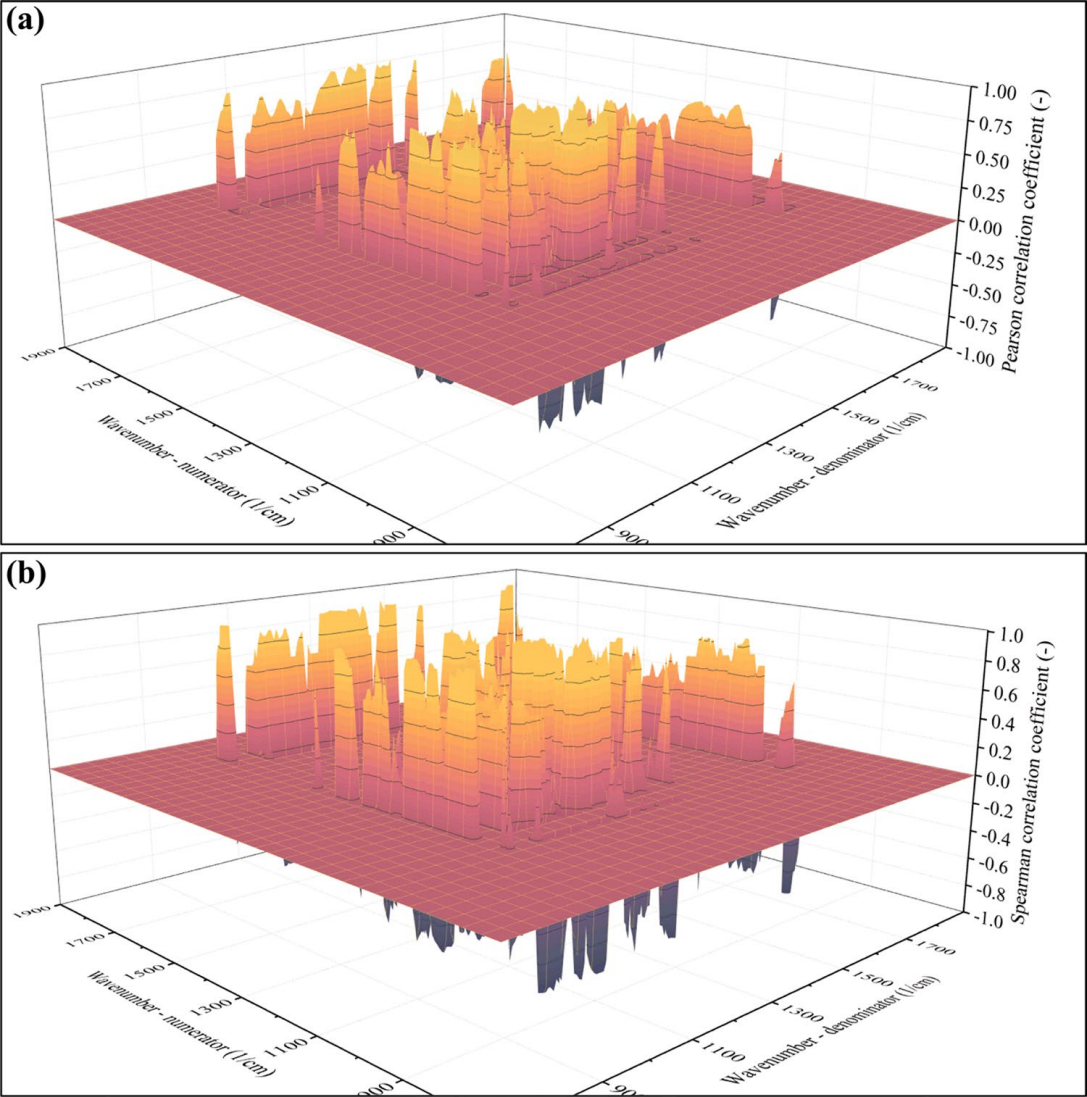
Values of the Pearson (**a**) and Spearman (**b**) correlation coefficients

So far, only the correlation between the average molecular weight (M_w_) and the ratio of absorbances at the A(1724 1/cm)/A(1278 1/cm) position has been investigated. Obviously, the ratios in other positions can also serve as the basis for modeling. The goal is to find all points on the surfaces exemplified in Fig. 4 that serve this purpose the best. A conventional method is the calculation of the correlation coefficient, often referred to as the Pearson correlation coefficient. The closer this coefficient is to + 1 (indicating a positive correlation) or -1 (indicating a negative correlation), the stronger the correlation between the variables. The stronger the correlation, the more accurate results the model will be able to provide. The Pearson correlation coefficients were calculated in the case of each absorbance pair in the 1900–700 1/cm range, leading to a surface function shown in Fig. 6a.

In the next step, points that exceed a certain threshold (*e.g.*, the absolute value of the correlation coefficient must be larger than 0.8) could be selected, and the model could be built on these points. While using this method is technically possible, it is disadvantageous for multiple reasons. Most importantly, the Pearson coefficient can describe linear correlation only and returns small values in the case of nonlinear tendencies, even if the variables are strongly correlated. As presented in Fig. 5, the empirical data points outline a nonlinear tendency, suggesting that this method might not be the best. To address the problem of nonlinearity, other types of correlation coefficients have also been proposed. Notable examples are rank-based correlation coefficients, such as the Spearman correlation coefficient (see Fig. 6b). Since the Spearman correlation coefficient can handle nonlinearity, it suits the data presented in Fig. 5 better than the Pearson correlation coefficient.

**Fig. 7:**
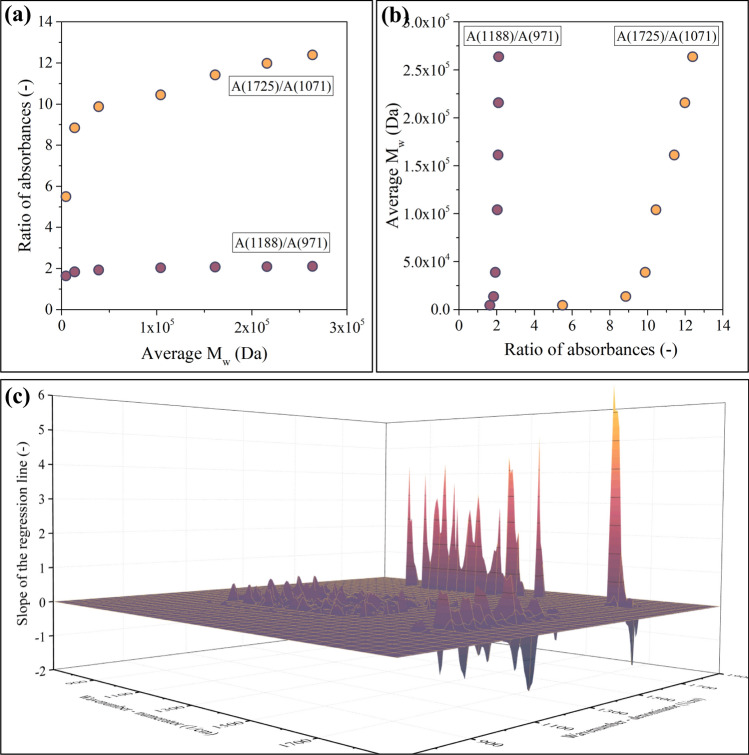
As opposed to the A(1725)/A(1071) data point, the A(1188)/A(971) ratio shows minimal dependence on M_w_ (**a**). This and similar data points lead to the appearance of extremely large slopes in *R*(*ν1*, *ν2*)—M_w_ plots, as presented in **b**. **c** Shows the results of multiplying the Spearman correlation coefficient with the slope of the regression line in *R*(*ν1*, *ν2*)—M_w_ plots, which is an effective way of addressing this problem

Unfortunately, the application of the Spearman correlation coefficient also has some disadvantages. The most important one is graphically presented in Fig. 7a, b.

Both data series plotted in Fig. 7a are extreme cases: all points are in increasing order; therefore, the Spearman correlation coefficient equals 1.0 in both cases. Even though the Spearman coefficient is the same, there is an important difference between the datasets. The absorbances recorded at 1188 1/cm and 971 1/cm are strongly correlated. In practice, this means that their ratio is about the same (~ 2), regardless of the molecular weight of the sample (Fig. 7a). Such regions cannot be used as a basis for building a model, as the dependent variable of the model can drastically change by only a slight change of the independent variable (Fig. 7b). Therefore, points such as the one in the A(1188 1/cm)/A(971 1/cm) position must be excluded.

Points such as the one in the A(1188 1/cm)/A(971 1/cm) position can be found (and subsequently excluded) in multiple ways. A simple and effective method is the calculation of the slope in the ratio of absorbances—average molecular weight plot (Fig. 7a). A near-zero slope indicates that such points will lead to models of poor performance. Therefore, this slope value is worth investigating in the case of each absorbance pair; see Fig. 7c. This diagram reveals that the slope visualized in Fig. 7a can be both positive and negative. As ANNs can handle both cases simultaneously, there is no need to limit the points included in the model to just one type of correlation. Simultaneously including regions where an increasing molecular weight leads to an increasing ratio of absorbances and regions where the tendency is the opposite makes the models more robust.

**Fig. 8:**
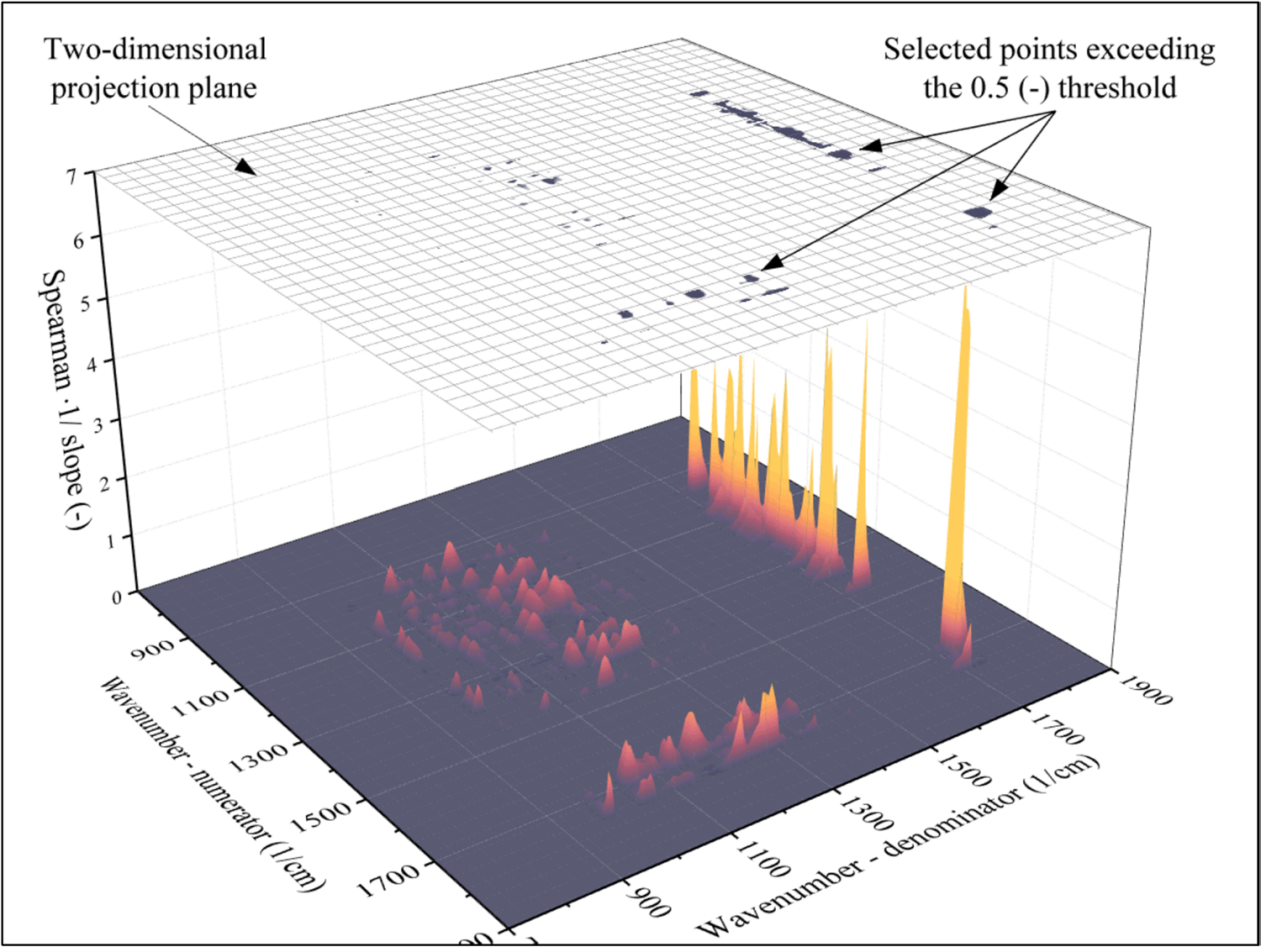
Surface function that indicates the suitability of the points to be used as independent variables for the model. If the indicator of suitability is larger than the threshold, the point will be used as an input (dark points in the two-dimensional projection), while the rest will be discarded (white area around the dark points in the two-dimensional projection)

In summary, two important factors must be considered while selecting the points that will be used as independent variables for the model. Firstly, the Spearman correlation coefficient (Fig. 6b) is worth multiplying by the slope of the ratio of absorbances—average molecular weight plot (Fig. 7c) because this step will eliminate the points that would make the model prone to minor perturbations. Second, the slope plotted in Fig. 7c can be both positive and negative. It is highly advantageous to include both types in the model. If the slope (Fig. 7c) is positive, the correlation coefficient (Fig. 6b) will also be positive. Similarly, if the slope is negative, the correlation coefficient will also be negative. Consequently, the product of these two indicators will always be positive and can be used as an indicator of suitability. The product of the Spearman correlation coefficient and the slope of the ratio of absorbances—average molecular weight plot is shown in Fig. 8.

The surface function shown in Fig. 8 can be considered an indicator of suitability. The larger the value, the better model can be built using the investigated point as an input for the neural network. Accordingly, points can be selected by determining a threshold. If the indicator of suitability exceeds the threshold (0.5 (–)), the point will be used as the independent variable for the model. The locations of points that have been selected with this thresholding method are marked using black in the two-dimensional projection plotted above the surface function in Fig. 8. The remaining points (white in the two-dimensional projection) cannot be used to build reliable models and will be discarded. As a result of this threshold-based filtering method, **148** absorbance ratios were selected. These points will serve as the input of the neural network, which will be discussed in the next section.

### Construction and training of the ANN

**Fig. 9:**
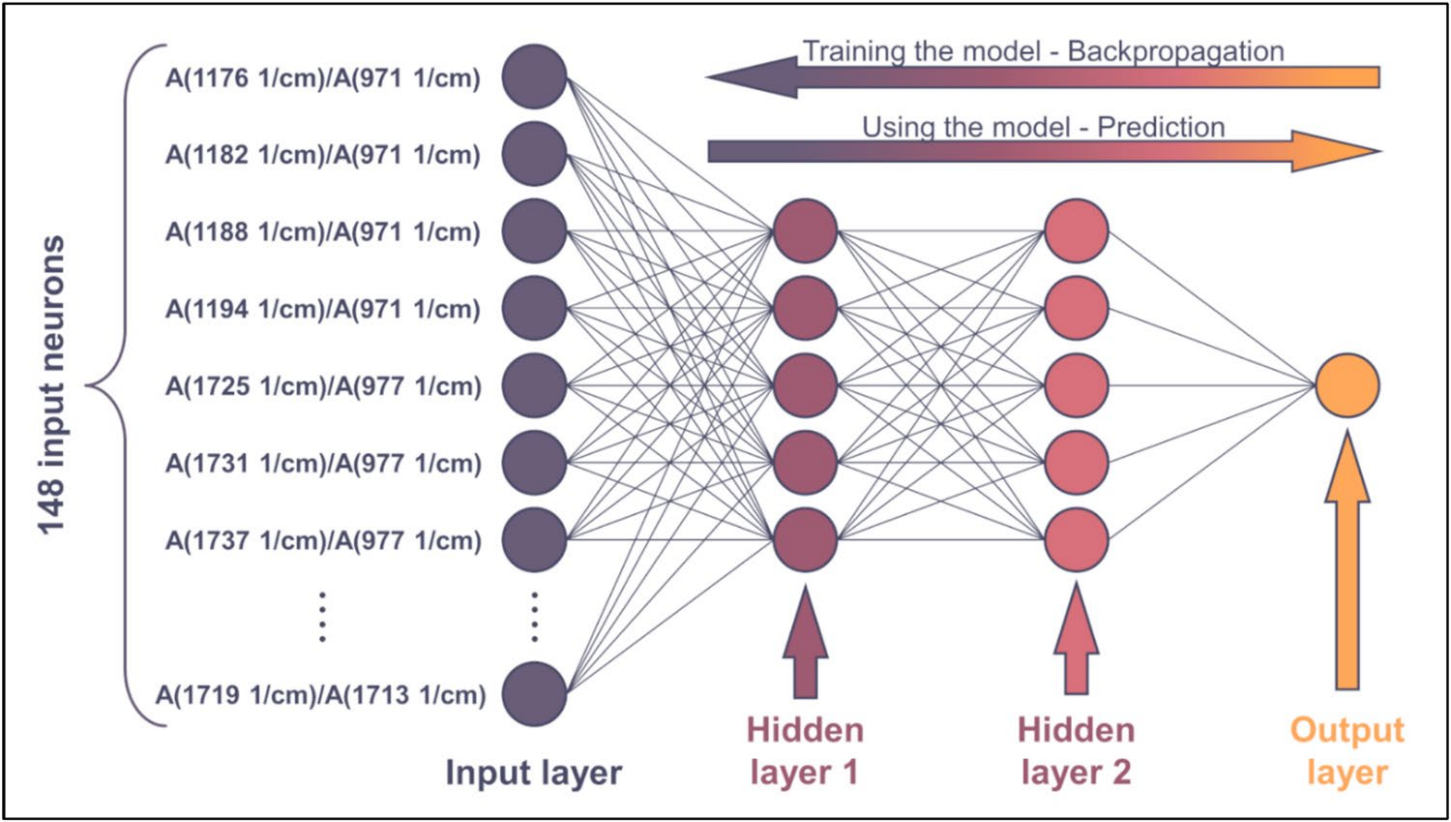
Scheme of the ANN. For the sake of simplicity, not all input neurons are drawn

The input of the neural network (Fig. 9) consists of 148 absorbances ratio—M_w_ data series that have been selected in the previous section. For the plot showing all these data series, please refer to the Supporting Information file SI1.

Since 148 points have been selected on the surface plotted in Fig. 8, the network has 148 input neurons (see Fig. 9). The subsequent layer in the network is the hidden layer. This layer does not need to consist of many neurons because the tendencies plotted in Fig. 5 are rather simple. As the activation function, a conventional sigmoid function will be used. The nonlinear characteristics of the regression curves in Fig. 5 can be approximated very well with a linear combination of only a few sigmoid functions. Therefore, increasing the number of neurons in the hidden layer beyond 3–5 is unnecessary and can lead to the problem of overparameterization. Likewise, increasing the number of hidden layers (deepening the network) is not expected to improve the accuracy of the calculation. Instead, it can lead to the problem of vanishing gradients, which makes training more difficult. Therefore, adding more than two hidden layers is not needed. Lastly, an output layer consisting of one neuron is to be added, as the model outputs one scalar (average M_w_). The ANN constructed in this way is shown in Fig. 9.

**Fig. 10:**
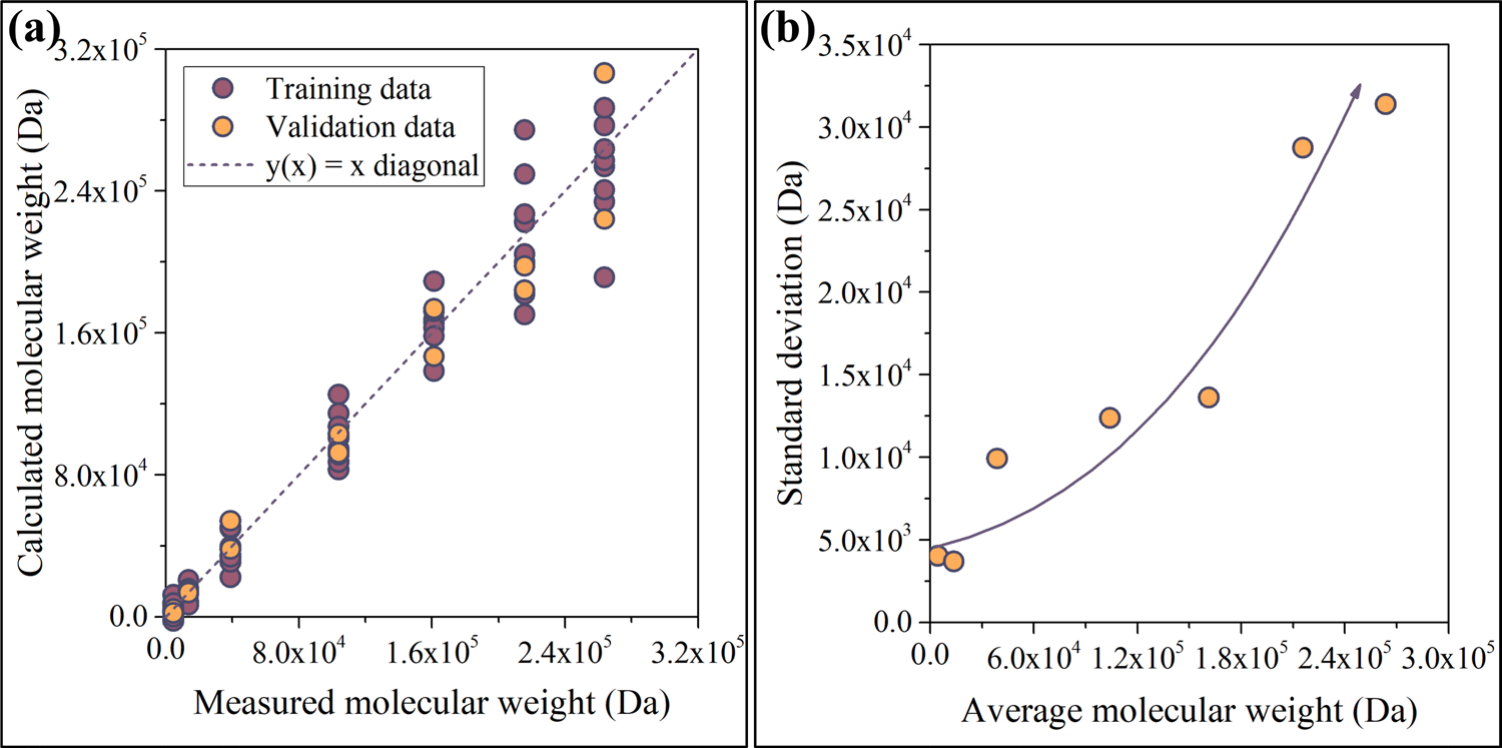
Performance of the model (**a**), and indicators of its accuracy (**b**). The model appears to be reliable below 200 kDa, but its accuracy deteriorates with increasing M_w_

The ANN has been trained by backpropagating the error of computation, see the arrow in the upper-right corner of Fig. 9. Backpropagation was based on the Levenberg–Marquardt algorithm. The network has been trained multiple times to gain information on the probability of convergent iteration. The training successfully converged in each test run (see Supporting Information file SI1). To ensure that the model had not been overparametrized, the available data was split. 80% of the spectra were used for training, whereas the remaining 20% were used for validation. Now, the network is ready to predict the average M_w_ of the samples. The results are plotted in Fig. 10a. Plotting the calculated values against their measured counterparts is a very effective method for visualizing the accuracy of the model. The purple and yellow circles in Fig. 10a represent training (80%) and validation data (20%), respectively.

As demonstrated in Fig. 10b, the model can provide predictions of sufficient accuracy below 200 kDa. The reduction of accuracy above 200 kDa can be ascribed to the saturation-like correlation between the average M_w_ and the ratio of absorbances; see Fig. 5a. Since most bacterial strains that were not subjected to genetic modification produce microbial polyesters with average molecular weights falling below 200 kDa, we can conclude that the model is able to provide accurate results for polyesters grades that bear industrial relevance.

## Conclusions

In this work, we have proposed a method enabling the calculation of the average molecular weight of microbial polyesters using FTIR data as input. The results presented in this paper demonstrate that the spectra are influenced by the average molecular weight of the samples, which can be a basis for building predictive machine learning models. By calculating and using absorbance ratios rather than absolute absorbances as input, our approach minimizes the impact of sample preparation inconsistencies, such as variations in film thickness, thereby enhancing the accuracy of the model. Points where absorbance ratios and molecular weights correlate closely were successfully found by using our new variable selection method based on the determination of correlation coefficients in combination with the calculation of the slope of regression curves depending on the average M_w_. Another benefit of using our approach is that the architecture of the neural network can be simultaneously tailored to suit the characteristics of the input data series and designed to prevent overparameterization. The plots showing indicators of accuracy have demonstrated that the trained ANN can output reliable results below 200 kDa. Therefore, we can conclude that most microbial polyester grades bearing industrial significance can be analyzed using the method proposed in this work.

## Supplementary Information

Below is the link to the electronic supplementary material.

Supplementary file1 (MP4 9232 KB)

Supplementary file2 (DOCX 1036 KB)

## Data Availability

This manuscript has data included as electronic supplementary material. Data will also be made available upon request.
