# Supplementary material for: Estimation of the average molecular weight of microbial polyesters from FTIR spectra using artificial intelligence
Source: Anal Sci. 2025 May 8;41(7):1015–27. doi: 10.1007/s44211-025-00780-2 (PMC12202672; doi:10.1007/s44211-025-00780-2)
Supplement: Supplementary file 2 — Supplementary file2 (DOCX 1036 KB) [file 44211_2025_780_MOESM2_ESM.docx]

**Supporting Information**

for the manuscript

**Estimation of the Average Molecular Weight of Microbial**

**Polyesters from Mid-FTIR Spectra using Artificial Intelligence**

Peter Polyak^1^*, Paweł Chaber^2^, Marta Musioł^2^, Grażyna Adamus^2^, Marek Kowalczuk^2^, Judit E. Puskas^1^, and Miroslawa El Fray^3,4^

^1^Department of Food, Agricultural and Biological Engineering, College of Food, Agricultural, and Environmental Sciences, The Ohio State University, 1680 Madison Avenue, Wooster, 44691, US

^2^Centre of Polymer and Carbon Materials, Polish Academy of Sciences, 34, M. Curie-Skłodowska St, Zabrze 41-819, Poland

^3^Department of Polymer and Biomaterials Science, West Pomeranian University of Technology in Szczecin, al. Piastow 45, 70-311 Szczecin, Poland

^4^Centre of Advanced Materials and Manufacturing Process Engineering, West Pomeranian University of Technology, Szczecin, al. Piastow 45, 70-311 Szczecin, Poland

*Corresponding author: Peter Polyak, polyak.10@osu.edu

**1. Processing spectra using Standard Normal Variate calculation**

**Fig. SI1** demonstrates the difference between the spectra that were not (**Fig. SI1a**) and were (**Fig. SI1b**) subjected to normalization using Standard Normal Variate (SNV) calculation. Since the differences between the absolute values are small, the SNV normalization does not change the appearance of the diagram considerably.


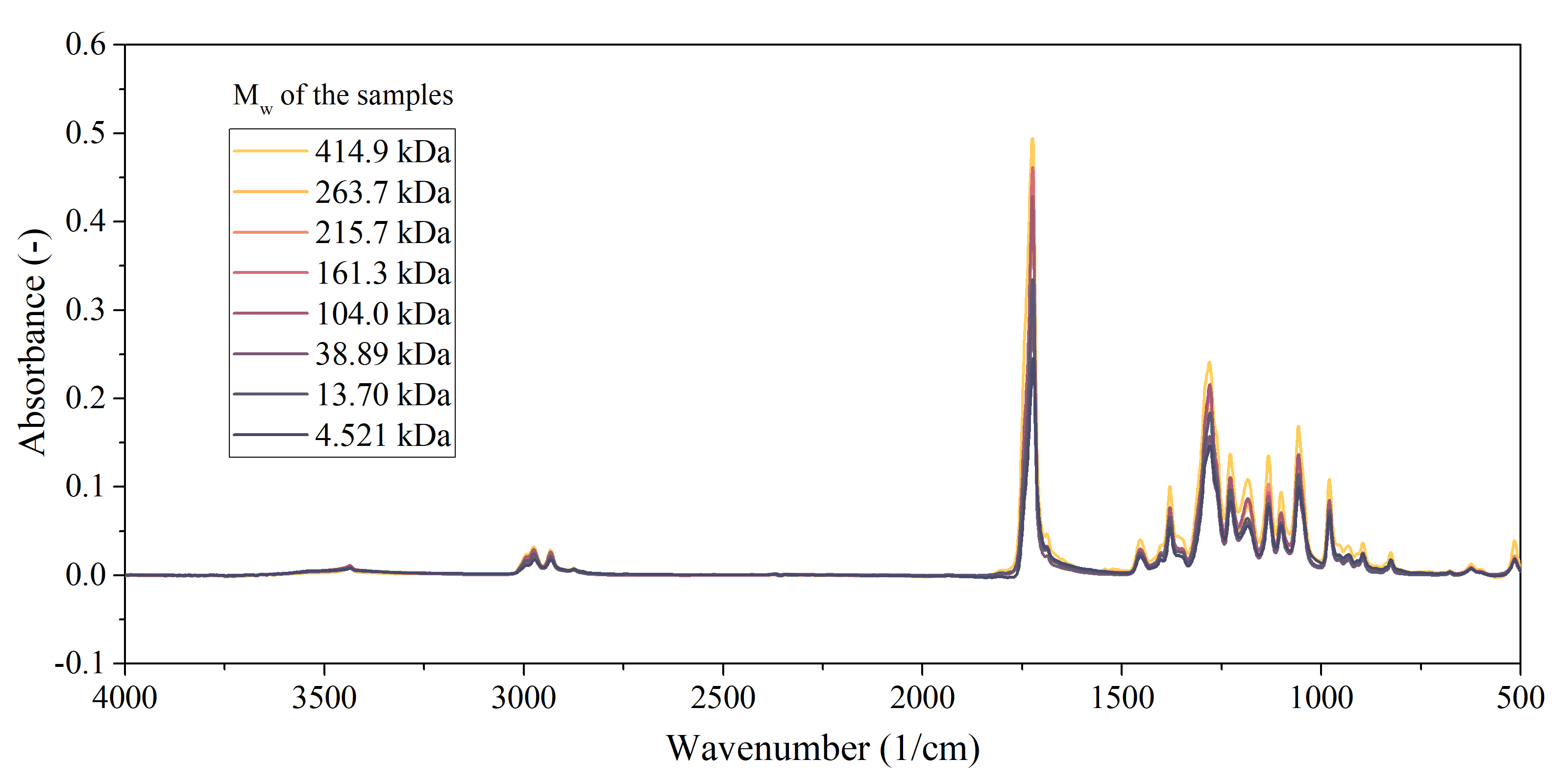


**(a)**

**(b)**


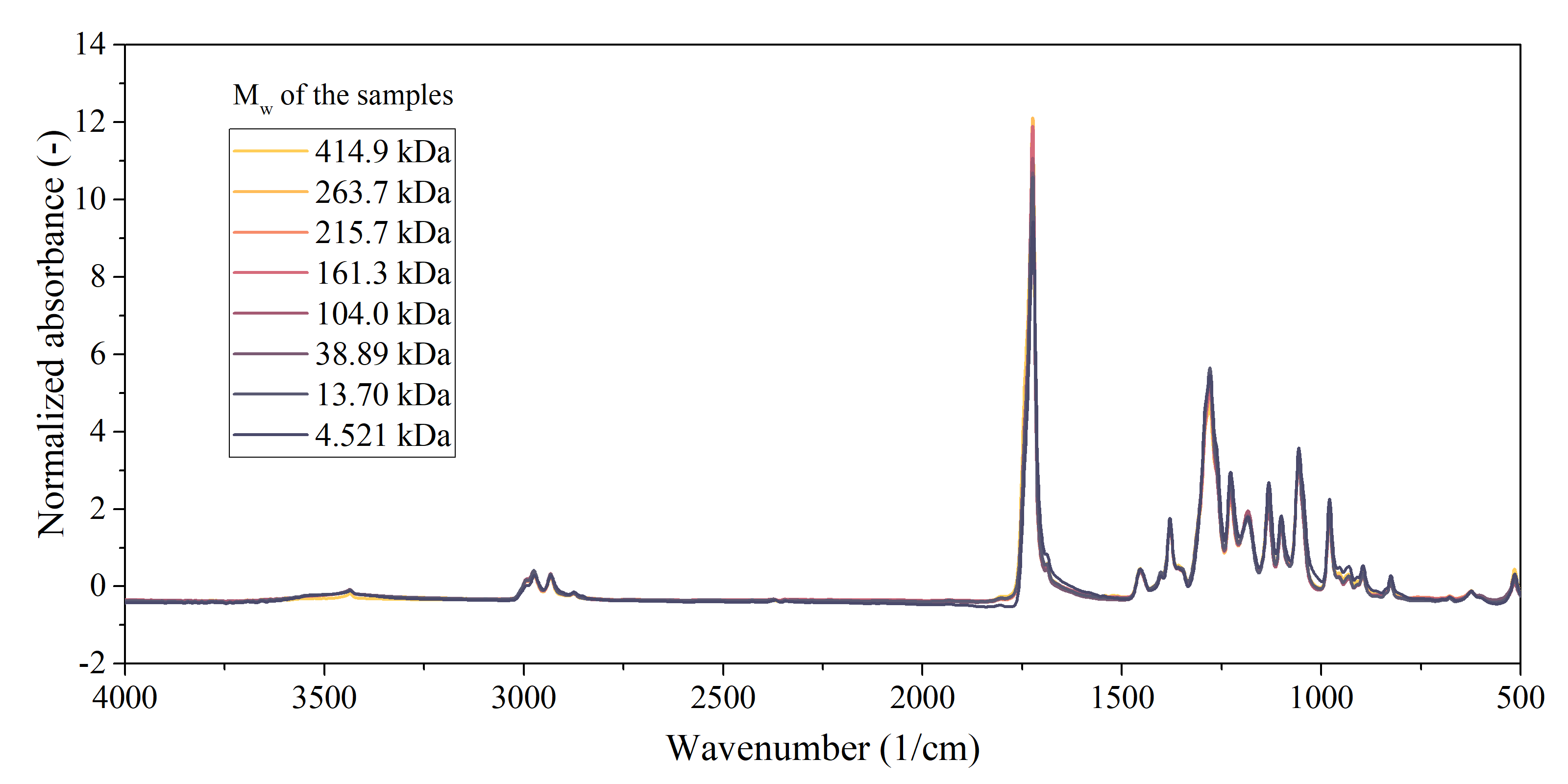


**Fig. SI1**: IR spectra that were not (**a**) and were (**b**) subjected to normalization using Standard Normal Variate (SNV) calculation.

In the next step, it is worth investigating whether the normalized absorbances outline a clear tendency between the amplitude of peaks and the average molecular weight. **Fig. SI2** demonstrates the normalized amplitude of the carbonyl peak plotted against the M_w_ of the sample. Although a weak positive correlation can be observed, the points appear to be biased by a considerable error. This finding suggests that modeling based on normalized absorbances is technically possible. However, as discussed in the paper, much clearer tendencies can be observed if the ratios of absorbances are calculated and plotted against the average molecular weight. Accordingly, the proposed model uses ratios of absorbances as input instead of normalized absorbances.


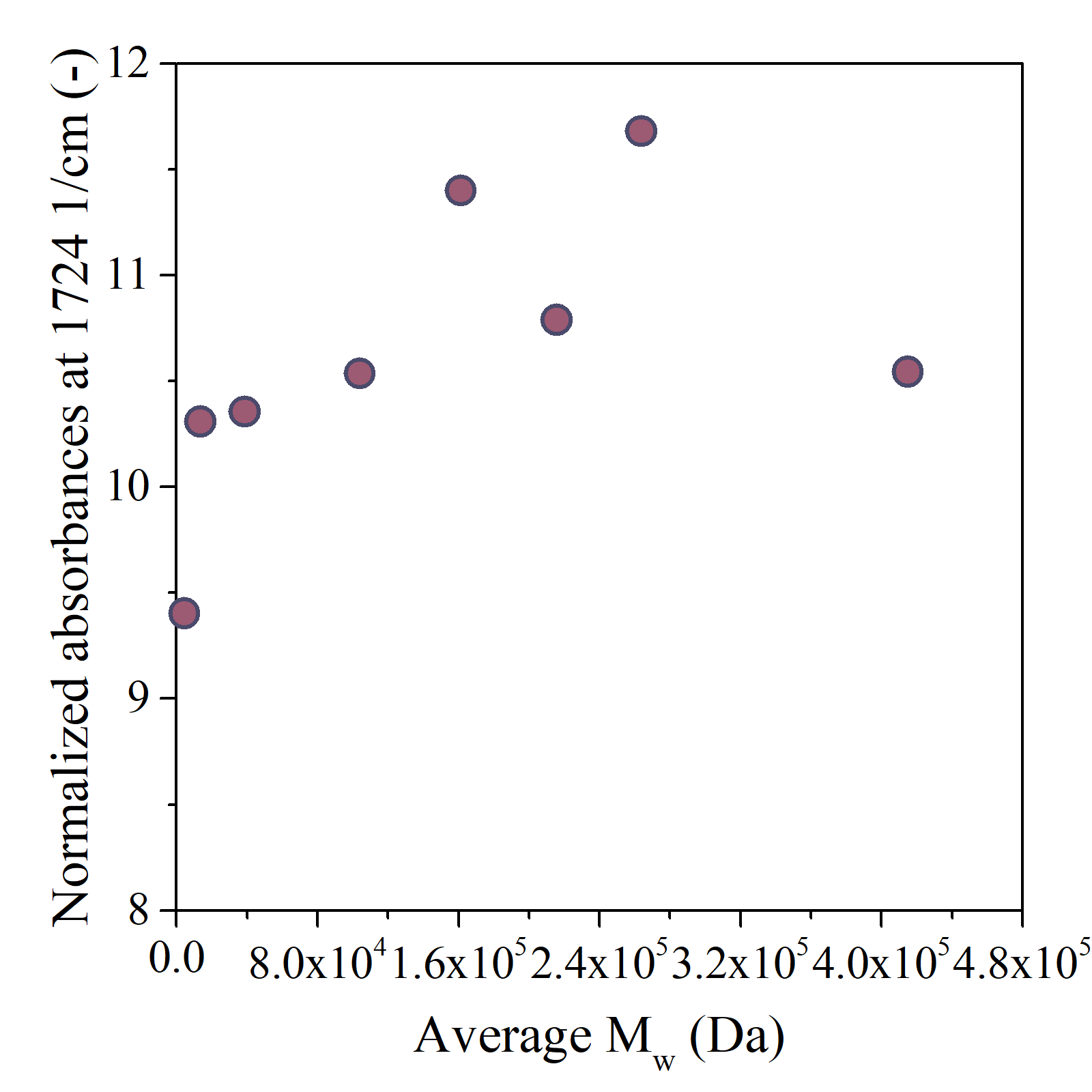


**Fig. SI2**: Normalized amplitudes of the carbonyl peak located at 1724 1/cm wavenumber.

**2. Designation of axes and the background of M_w_-dependent accuracy**

The experimental work consists of the creation and analysis of samples with different average molecular weights; therefore, plotting this parameter on the horizontal axis (as an independent variable) appears to be a convenient and straightforward way of visualizing the data (see **Fig. SI3a**). However, the model will use the ratios of absorbances as the independent variable. Therefore, in the context of predictive models that use spectral data to determine the M_w_, the independent variable is the ratio of absorbances, whereas the dependent variable is the average molecular weight. Accordingly, the former is to be plotted on the horizontal axis, and the latter is to be plotted on the vertical axis. The diagram prepared in this way is shown in **Fig. SI3b**.

**(b)**

**(a)**


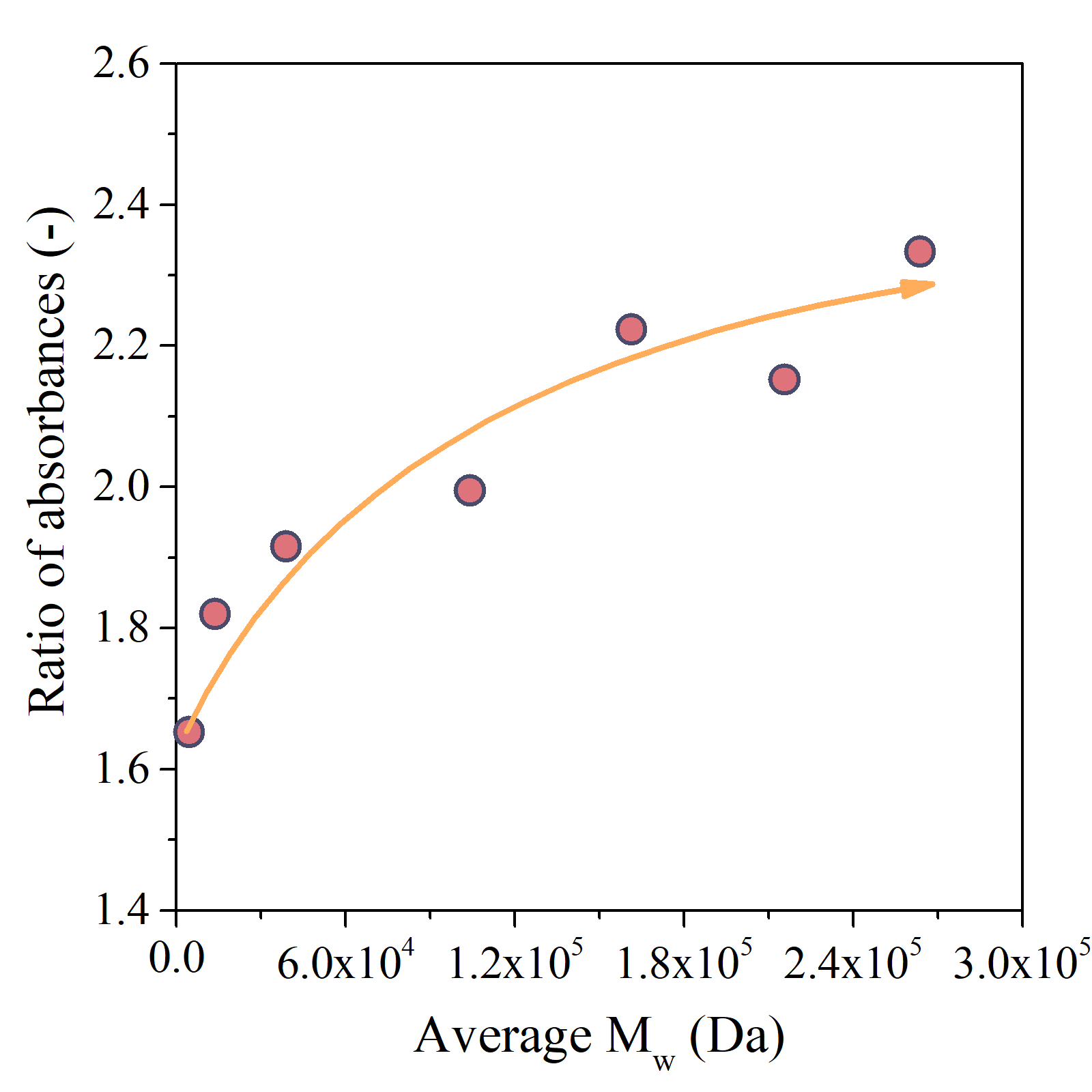

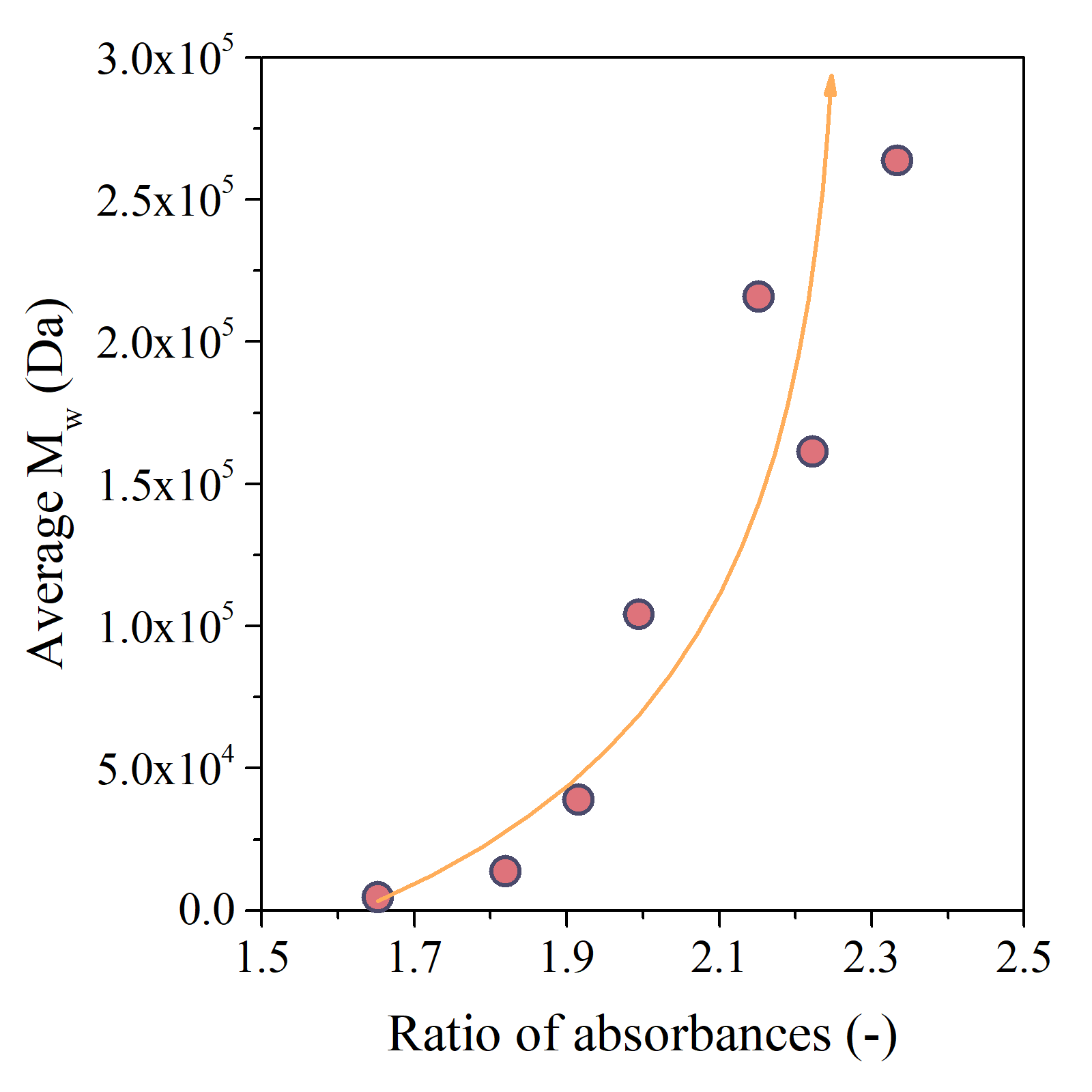


**Fig. SI3**: Ratio of absorbances (A_1724_/A_1278_) plotted against the average molecular weight (**a**) and its inverse that will be a basis for modeling (**b**). As the regression curve of the latter converges to infinity, the model is expected to become less accurate at high M_w_ values.

**Fig. SI3b** sheds light on an important and nonnegligible limitation of this approach. Since the method relies on the influence of end groups, it works well if the relative amount of end groups is large. However, the longer the macromolecules, the less the end groups influence the spectrum. Therefore, at large average molecular weights, the effect of the end groups is small, and increasing the molecular weight even further barely affects the characteristics of the spectral peaks. Therefore, if absorbance ratios are plotted as a function of molecular weights, the points will outline saturation-like characteristics (**Fig. SI3a**). In comparison, if the molecular weight is plotted as a function of absorbance ratios, the regression curve will converge to infinity (**Fig. SI3b**), suggesting that the larger the M_w_ and the larger the slope of the regression curve exemplified in **Fig. SI3b**, the less reliable the model becomes. Nevertheless, in the molecular weight region bearing practical relevance (10 000 - 200 000 Da), the slope of the regression curve in **Fig. SI3b** is rather small, enabling a calculation of M_w_s with acceptable accuracy.

**3. Graphical representation of the indicator of suitability**

As discussed in the paper, calculating and plotting the product of the Spearman correlation coefficient and the slope of the ratio of absorbances - M_w_ correlation is an effective method of quantifying the suitability of the investigated point to serve as an input for the model. Therefore, this product can be considered an indicator of suitability: the larger the value, the more reliable the model becomes. The surface function consisting of all calculated points is presented in **Fig. SI4a**. The goal is to select the regions where the indicator of suitability is the largest. These regions appear as peaks in **Fig. SI4a**. A color-coded, two-dimensional projection of the suitability function is shown in **Fig. SI4b**. Geometrically, **Fig. SI4b** is the top view of the surface function shown in **Fig. SI4a**. Selecting points on this surface can be based on setting a limit/threshold: if the indicator of suitability is larger than 0.5, the point will be selected as the independent variable for the model (dark points in **Fig. SI4c**), while the remaining points (white areas in **Fig. SI4c**) will be discarded.


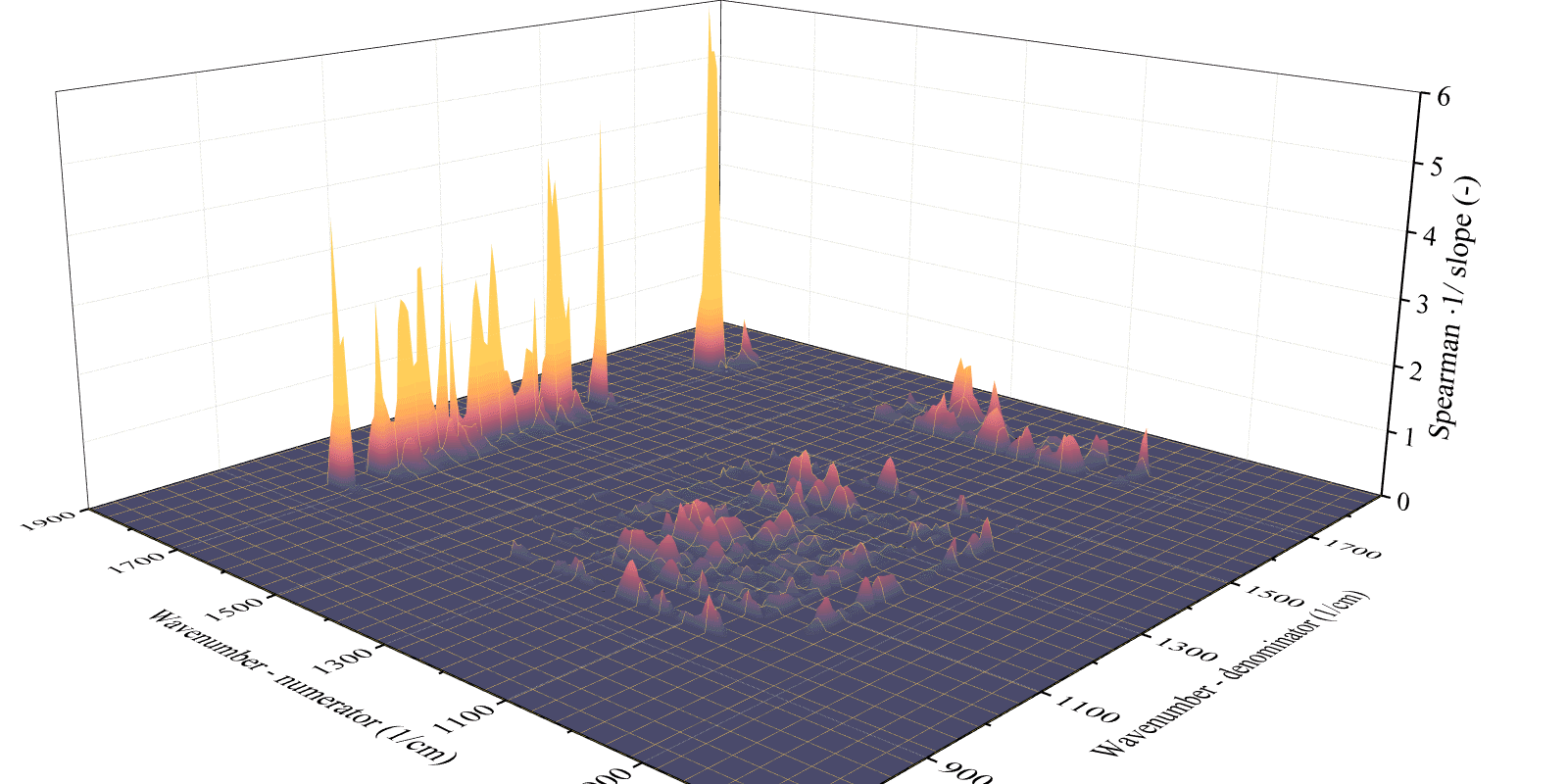


**(c)**

**(b)**

**(a)**


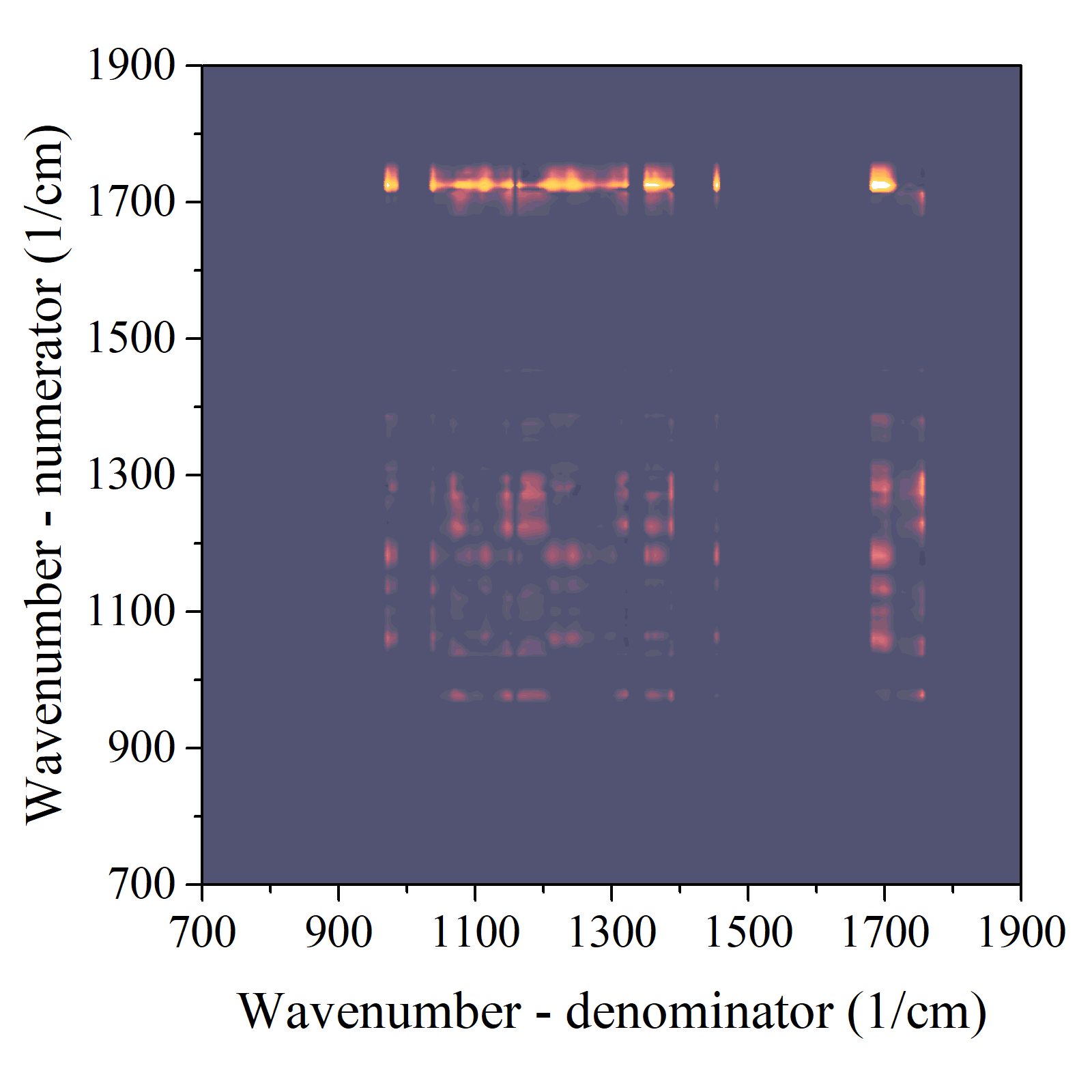

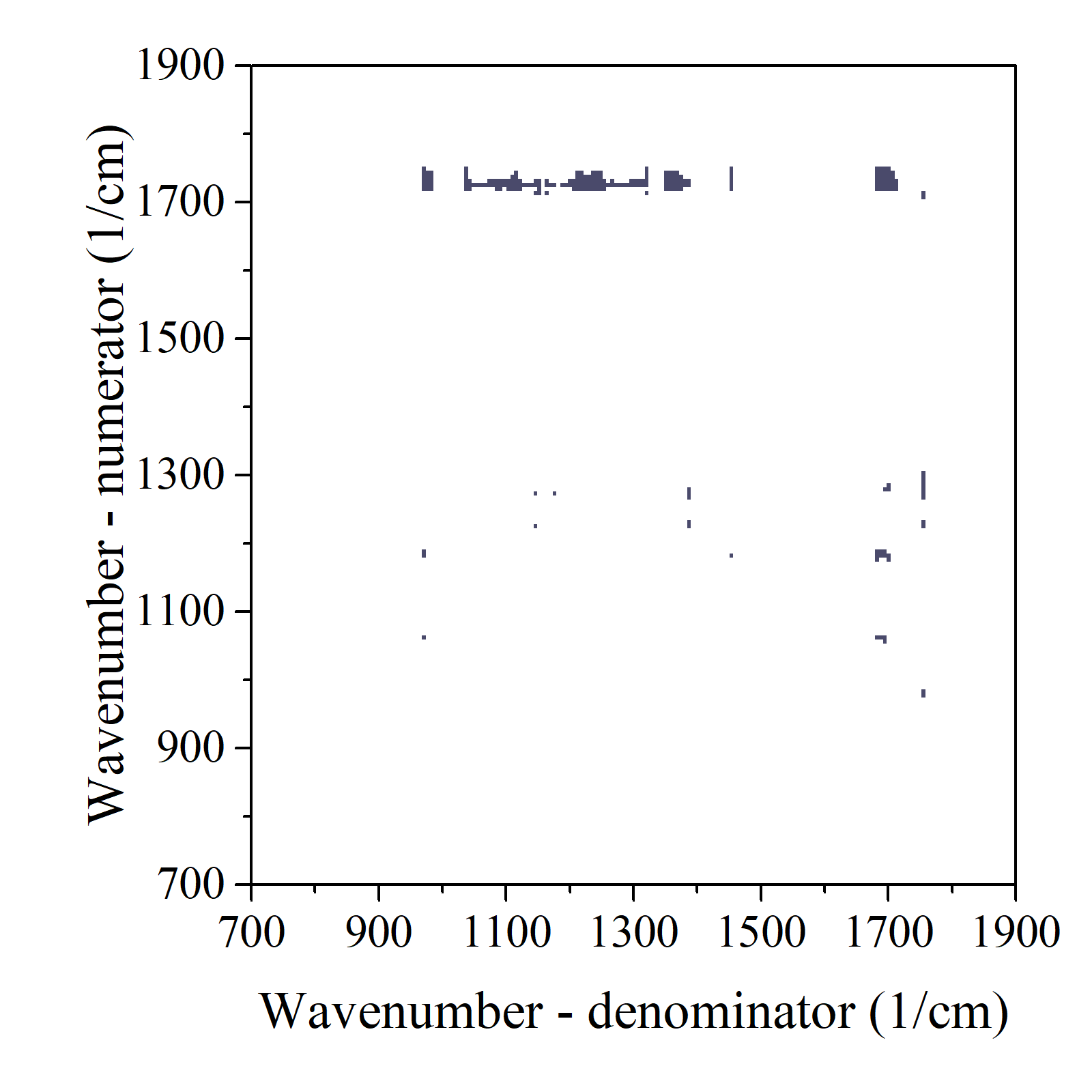


**Fig. SI4**: Surface function that indicates the suitability of the points to be used as independent variables for the model (**a**). Figure (**b**) shows the two-dimensional projection of this three-dimensional surface. If the indicator of suitability (**b**) is larger than the threshold (0.5 (-)), the point will be used as an input for the model (dark points in **c**), while the rest will be discarded (white area around the dark points in **c**).

**4. Plots of all selected data series**

As a result of the threshold-based filtering method, 148 individual points have been selected (the dark areas in **Fig. SI4c** consist of 148 points). Since experiments were carried out using a sequence of seven samples, each selected point represents a sequence of seven M_w_-absorbance ratio data pairs. These data sequences, serving as an input for the model, are plotted in **Fig. SI5a**. As the ratios are varied in a rather wide interval, individual correlations are easier to observe if the plot is normalized (**Fig. SI5b**). Out of the 148 data sequences, the correlation is positive in 121 cases and negative in 27 cases. ANNs are very flexible and can handle positive and negative correlations simultaneously. To leverage this flexibility, all 148 data sequences are included in the training dataset.

**(b)**

**(a)**


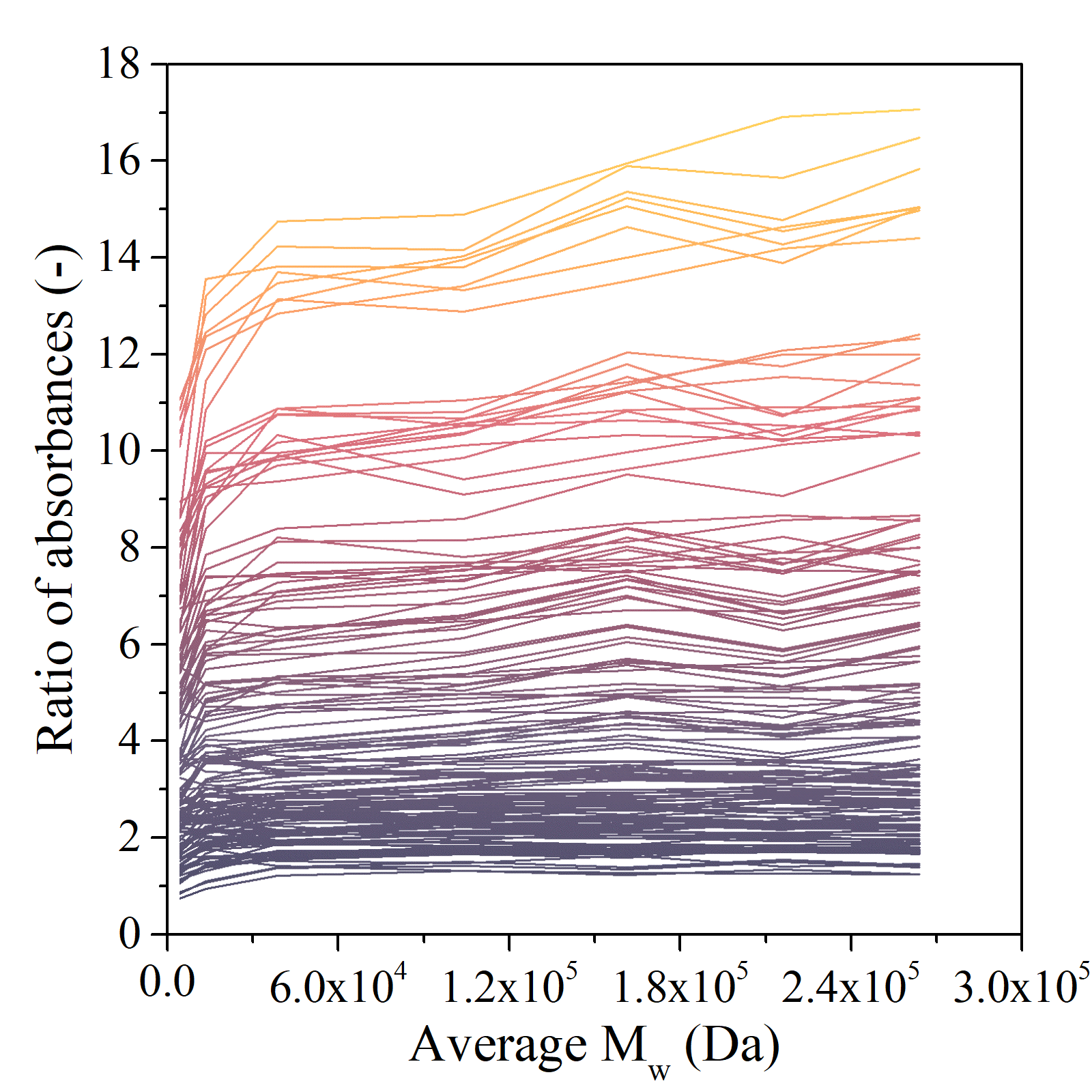

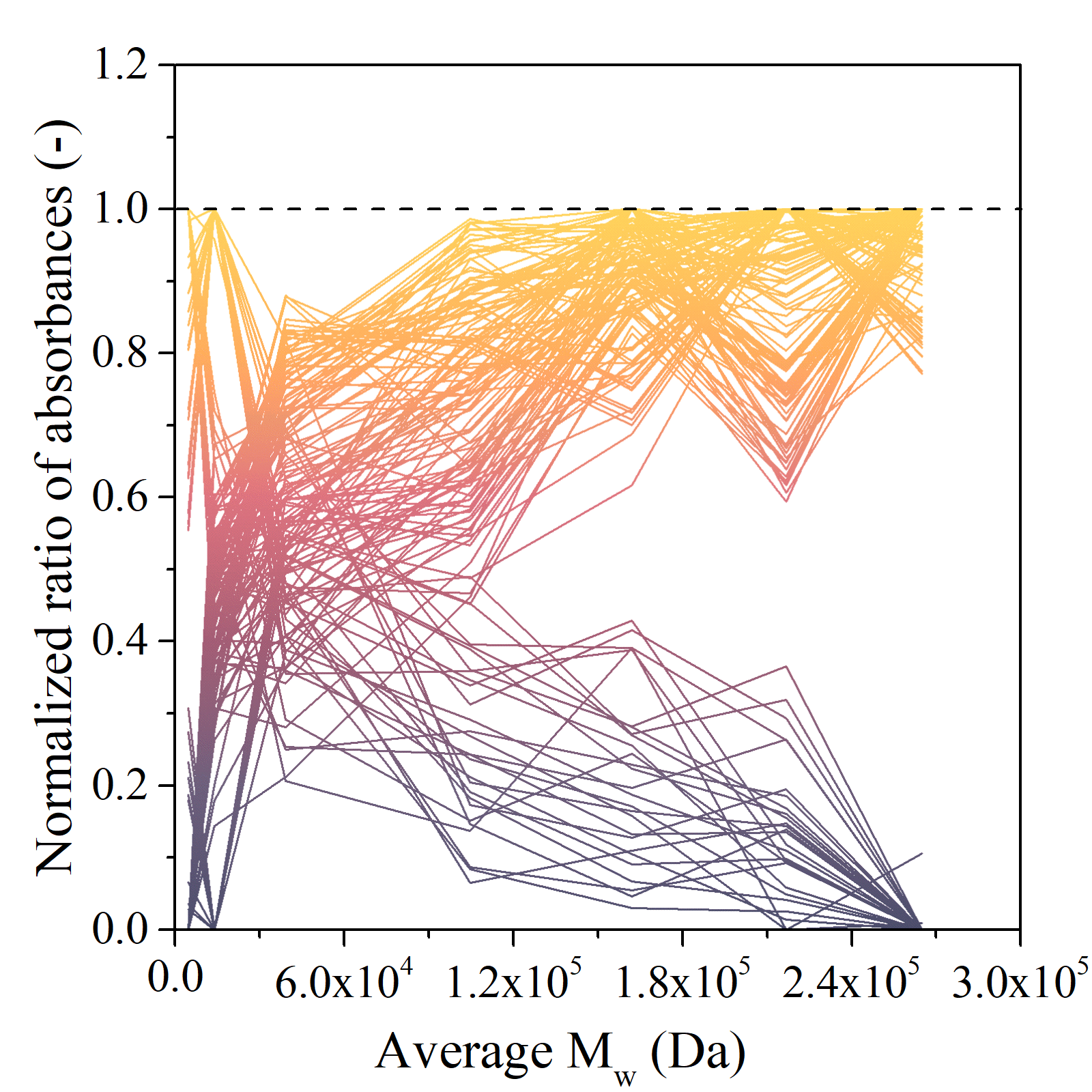


**Fig. SI5**: All the selected ratio of absorbances - M_w_ values (**a**) and the result of their normalization (**b**). The data in (**a**) and (**b**) is the input for the neural network.

**5. Learning curves**

The network has been trained multiple times to gain information on the probability of convergent iteration. The training successfully converged in each test run, as presented in **Fig. SI6**. A training epoch (independent variable of **Fig. SI6**) is one iteration cycle in which all the training data is backpropagated in the network. The plot shows the performance of the model on both training and validation data. Splitting the available data into training and validation subsets is an effective way of addressing the problem of overparameterization. An overparametrized model performs well on the data that was used to train the model but fails to provide accurate results for input that was not part of the training dataset. **Fig. SI6** highlights that the model performs similarly on both training and validation data, which proves that no overparameterization took place.


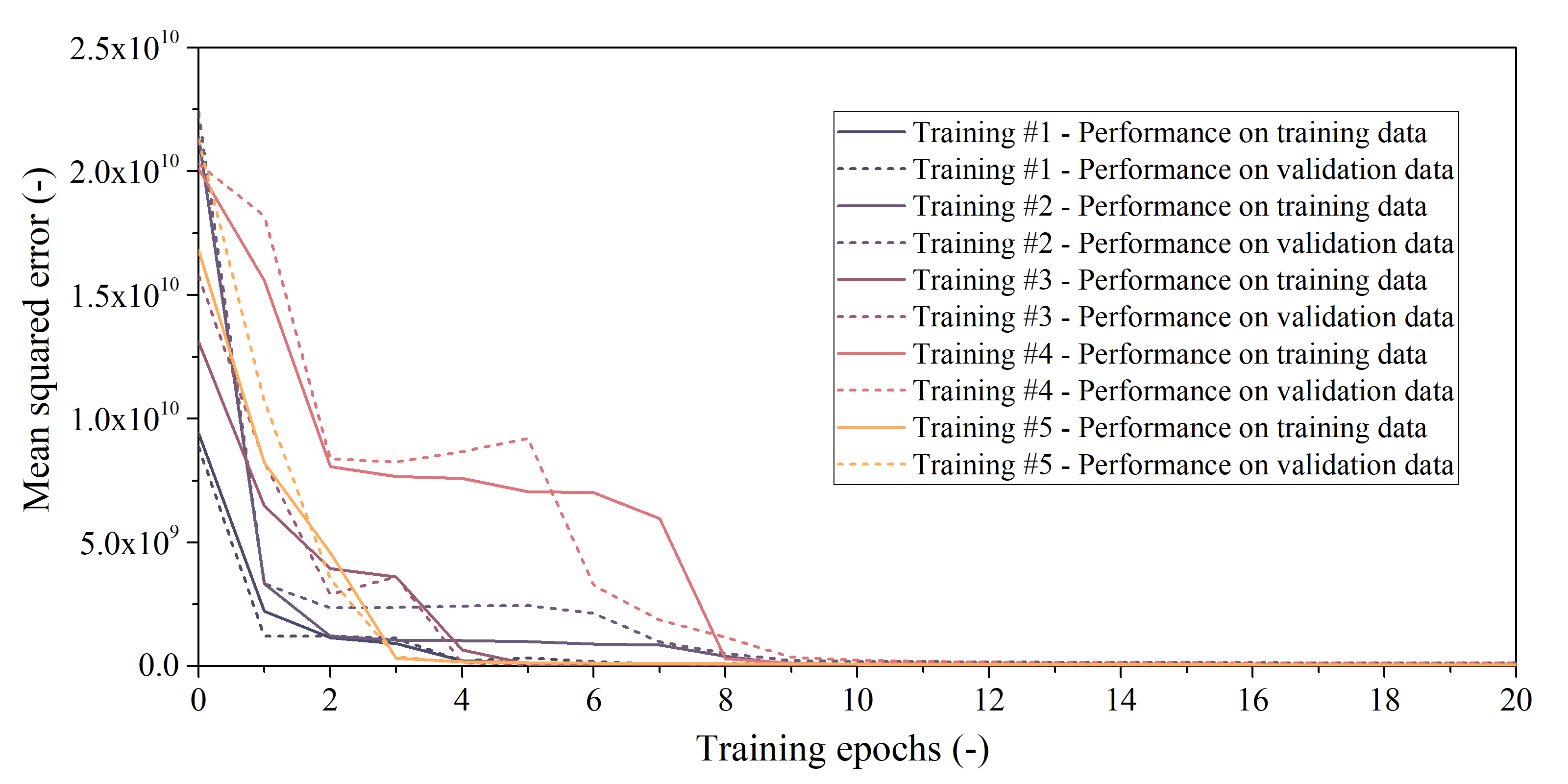


**Fig. SI6**: Learning curves indicating that the model has not been overparametrized.
